# Crystal structure of *trans*-bis­{4-bromo-*N*-[(pyridin-2-yl)­methyl­idene]aniline-κ^2^ *N*,*N*′}di­chlorido­ruthenium(II)

**DOI:** 10.1107/S205698901501556X

**Published:** 2015-08-22

**Authors:** Kittipong Chainok, Filip Kielar

**Affiliations:** a Department of Chemistry, Faculty of Science, Naresuan University, Muang, Phitsanulok, 65000, Thailand

**Keywords:** crystal structure, Schiff base ligand, π–π stacking, ruthenium(II)

## Abstract

In the title complex, [RuCl_2_(C_12_H_9_BrN_2_)_2_] or [RuCl_2_(PM-BrA)_2_] (PM-BrA = 4-bromo-*N*-(2′-pyridyl­methyl­ene)aniline), the Ru^II^ cation is located on a centre of inversion and is surrounded by four N atoms of two PM-BrA ligands in the equatorial plane and by two Cl atoms in a *trans* axial arrangement, displaying a distorted octa­hedral coordination environment. Two C atoms in the benzene ring of the PM-BrA ligand are equally disordered over two sets of sites. The benzene and pyridine rings of the PM-BrA ligand are oriented at dihedral angles of 62.1 (10) and 73.7 (11)° under consideration of the two orientations of the disordered benzene ring. In the crystal, the complex mol­ecules are connected *via* C—H⋯Cl hydrogen-bonding inter­actions into a layered arrangement parallel (100). C—H⋯Br hydrogen bonding and weak aromatic π–π stacking inter­actions complete a three-dimensional supra­molecular network.

## Abstract 2

The Ru^II^ atom in the title complex is surrounded by a distorted Cl_2_N_4_ coordination set. In the crystal structure, adjacent complex mol­ecules are connected through C—H⋯Cl hydrogen-bonding inter­actions into a layered arrangement parallel to (100). Additional C—H⋯Br hydrogen-bonding inter­actions along with π–π stacking inter­actions complete a three-dimensional supra­molecular network.

## Chemical context   

Bidentate Schiff bases are one of the most widely used ligands in coordination chemistry. Their complexes have found utility in a wide range of applications (Rezaeivala & Keypour, 2014 ▸; Gupta & Sutar, 2008 ▸). In particular, ruthenium(II) complexes of Schiff bases have been shown to display a variety of structural features and exhibit inter­esting biological and catalytic reactivities (Li *et al.*, 2015 ▸; Wang *et al.*, 2015 ▸; Drozdzak *et al.*, 2005 ▸). Herein, we report the synthesis and crystal structure of a ruthenium(II) complex with the bidentate Schiff base ligand of 4-bromo-*N*-(2′-pyridyl­methyl­ene)aniline (PM-BrA), [RuCl_2_(C_12_H_9_BrN_2_)_2_], (I).
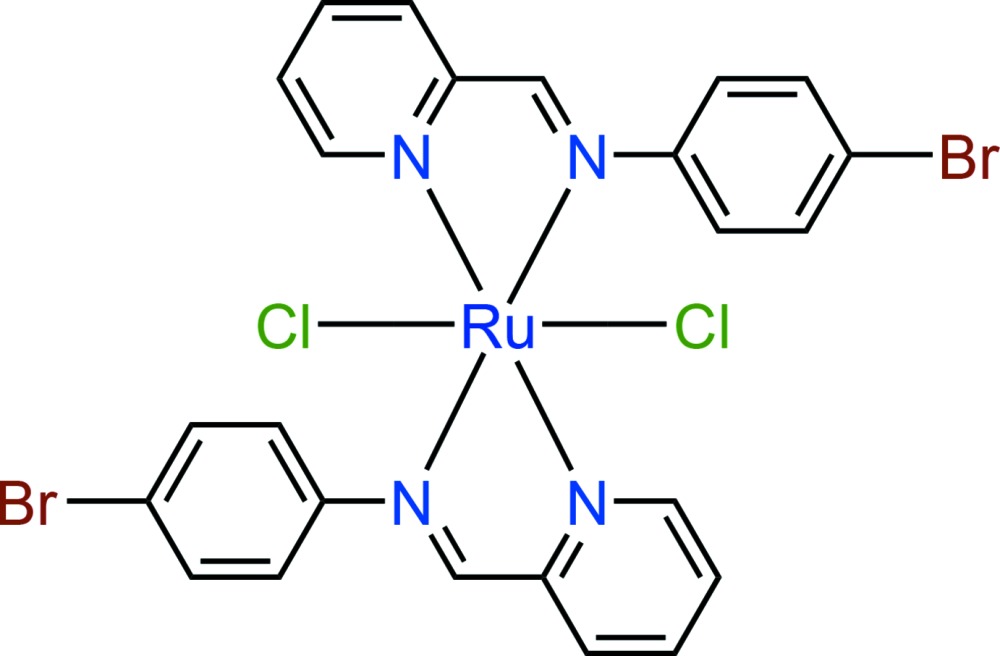


## Structural commentary   

**Figure 1:**
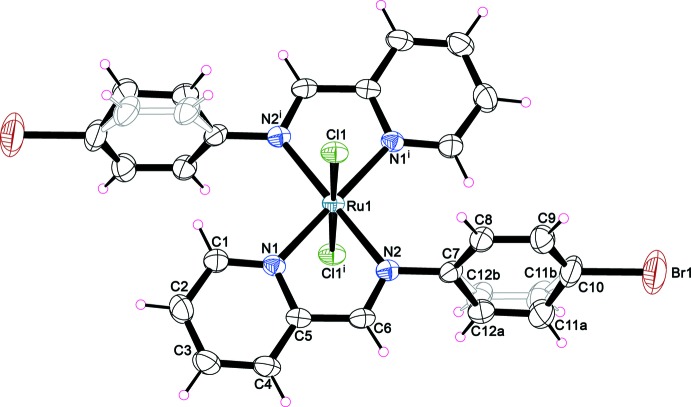
The mol­ecular structure of complex (I), showing displacement ellipsoids at the 50% probability level. Disorder is displayed for the C11 and C12 atoms of the benzene ring. [Symmetry operator: (i) −*x* + 1, −*y* + 1, −*z*.]

The asymmetric unit of compound (I) contains one half of the complex mol­ecule with the Ru^II^ cation lying on an inversion centre (Fig. 1 ▸). The coordination environment around Ru^II^ is a distorted [Cl_2_N_4_] octa­hedron, whereby the metal is chelated by two PM-BrA ligands in the equatorial plane and by two Cl atoms in a *trans* axial arrangement. The ligand exhibits an N1⋯N2 bite distance of 2.585 (7) Å with an N1—Ru1—N2 bite angle of 76.9 (1)°. The reduced bite angle of the chelating ligand is one of the main factors accounting for the distortion from the ideal octa­hedral geometry of the coordination polyhedron, with the the largest *cis* angle being 103.1 (2)°. The Ru—N bond lengths are 2.073 (5) and 2.084 (5) Å, and the Ru—Cl bond length is 2.3908 (14) Å, in agreement with those observed in the structures of similar compounds (Roy *et al.*, 2012 ▸). Two C atoms in the benzene ring of the PM-BrA ligand are equally disordered over two sets of sites. The dihedral angle between the least-square planes of the benzene and pyridine rings in the PM-BrA ligand are 62.1 (10) and 73.7 (11)° under consideration of the two orientations of the disordered benzene ring.

## Supra­molecular features   

**Figure 2:**
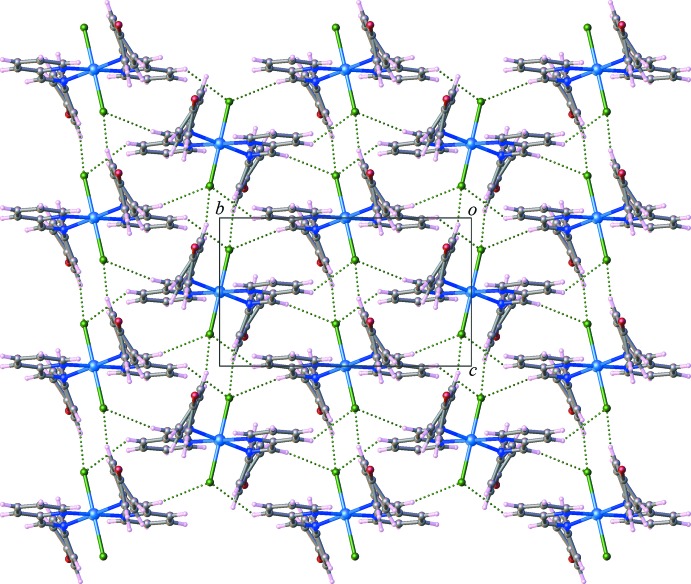
Crystal packing of complex (I) in a view along [100]. C—H⋯Cl hydrogen-bonding inter­actions are shown as dashed lines.

**Figure 3:**
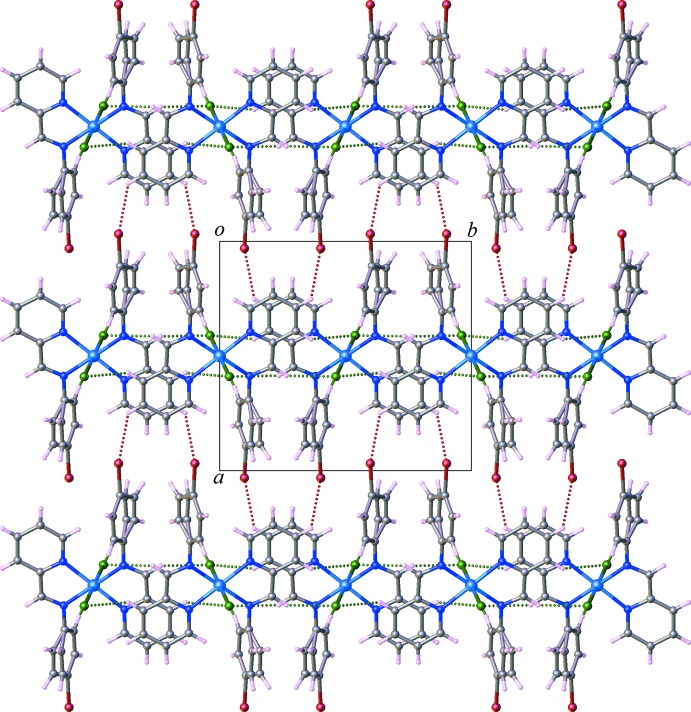
Crystal packing and C—H⋯Br and C—H⋯Cl hydrogen-bonding inter­actions (dashed lines) in complex (I), viewed along [001].

In the crystal, weak inter­molecular C—H⋯Cl hydrogen-bonding inter­actions between the C atoms of the benzene ring and the Cl atoms connect the complex mol­ecules into a supra­molecular layered arrangement parallel to (100) (Fig. 2 ▸). As shown in Fig. 3 ▸, a C—H⋯Br hydrogen bond between the phenyl C atoms and the Br atoms, along with weak aromatic π–π stacking inter­actions [centroid-to-centroid distance = 4.107 (4) Å, dihedral angle = 0.7 (3)°] complete a three-dimensional supra­molecular network. Numerical values of C—H⋯*X* (*X* = Cl, Br) inter­actions are compiled in Table 1 ▸.

## Database survey   

The structure of *trans*-[RuCl_2_(Hpyrimol)_2_] (Hpyrimol = 4-methyl-2-*N*-(2-pyridyl­methyl­ene)amino­phenol) with a closely related Schiff base N_2_ donor set for each ligand has been reported (Roy *et al.*, 2012 ▸). The bond lengths and bond angles in this complex are in agreement with those in the structure of (I). A search of the Cambridge Structural Database (Version 5.36, last update February 2015; Groom & Allen, 2014 ▸) gave 12 hits for complexes involving transition metals and the ligand PM-BrA (KISZIX, KISZOD, KISZUJ, Davies *et al.*, 2014 ▸; XEDCUG, Khalaji *et al.*, 2012 ▸; UNIZOH, Harding *et al.*, 2011 ▸; SUYDAS, Harding *et al.*, 2010 ▸; FOWBOJ, Khalaj *et al.*, 2009 ▸; FOWBID, Mahmoudi *et al.*, 2009 ▸; MOYDUA, Dehghanpour *et al.*, 2009 ▸; TULKIV, Gao *et al.*, 2009 ▸; YOCZAS, Khalaj *et al.*, 2008 ▸; YOCZEW, Mahmoudi *et al.*, 2008 ▸).

## Synthesis and crystallization   

A solution of the ligand 4-bromo-*N*-(2′-pyridyl­methyl­ene)aniline (104.4 mg, 0.4 mmol) in dry methanol (5 ml) was placed in a test tube. A solution of RuCl_3_ (41.5 mg, 0.2 mmol) in dry methanol (5 ml) was then carefully layered on the top of a methano­lic solution. After slow diffusion at room temperature for three days, pale-green plate- or block-like crystals of complex (I) were obtained.

## Refinement   

Crystal data, data collection and structure refinement details are summarized in Table 2 ▸. Hydrogen atoms were positioned with idealized geometry and refined with *U*_iso_(H) = 1.2*U*_eq_(C) using a riding model with C—H = 0.95 Å. C atoms C11 and C12 and attached H atoms in the benzene ring are disordered over two set of sites and were refined using a split model with equal occupancy.

## Supplementary Material

Crystal structure: contains datablock(s) I. DOI: 10.1107/S205698901501556X/wm5204sup1.cif

Structure factors: contains datablock(s) I. DOI: 10.1107/S205698901501556X/wm5204Isup2.hkl

Supporting information file. DOI: 10.1107/S205698901501556X/wm5204Isup3.cdx

CCDC reference: 1419653

Additional supporting information:  crystallographic information; 3D view; checkCIF report

## Figures and Tables

**Table 1:** Hydrogen-bond geometry (, )

*D*H*A*	*D*H	H*A*	*D* *A*	*D*H*A*
C8H8Cl1^i^	0.93	2.79	3.472(7)	132
C6H6Cl1^ii^	0.93	2.83	3.673(7)	151
C3H3Br1^iii^	0.93	3.13	3.797(8)	131
C4H4Cl1^iv^	0.93	2.94	3.529(7)	122

Symmetry codes: (i) 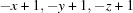; (ii) 
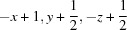
; (iii) 
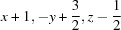
; (iv) 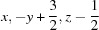.

**Table 2:** Experimental details

Crystal data	
Chemical formula	[RuCl_2_(C_12_H_9_BrN_2_)]
*M*_r_	694.21
Crystal system, space group	Monoclinic, *P*2_1_/*c*
Temperature (K)	296
*a*, *b*, *c* ()	12.3270(7), 13.3114(7), 7.9673(4)
()	100.091(2)
*V* (^3^)	1287.13(12)
*Z*	2
Radiation type	Mo *K*
(mm^1^)	3.94
Crystal size (mm)	0.26 0.20 0.18

Data collection	
Diffractometer	Bruker APEXII CCD
Absorption correction	Multi-scan (*SADABS*; Bruker, 2014 ▸)
*T*_min_, *T*_max_	0.549, 0.745
No. of measured, independent and observed [*I* > 2(*I*)] reflections	15951, 2391, 1844
*R*_int_	0.054
(sin /)_max_ (^1^)	0.607

Refinement	
*R*[*F* ^2^ > 2(*F* ^2^)], *wR*(*F* ^2^), *S*	0.054, 0.152, 1.04
No. of reflections	2391
No. of parameters	170
No. of restraints	73
H-atom treatment	H-atom parameters constrained
_max_, _min_ (e ^3^)	0.96, 1.29

Computer programs: *APEX2* and *SAINT* (Bruker, 2014 ▸), *SHELXT* (Sheldrick, 2015*a* ▸), *SHELXL2007* (Sheldrick, 2015*b* ▸), *OLEX2* (Dolomanov *et al.*, 2009 ▸), *publCIF* (Westrip, 2010 ▸) and *enCIFer* (Allen *et al.*, 2004 ▸).

## supplementary crystallographic information

### Crystal data

**Table:**

[RuCl_2_(C_12_H_9_BrN_2_)]	*F*(000) = 676
*M**_r_* = 694.21	*D*_x_ = 1.791 Mg m^−^^3^
Monoclinic, *P*2_1_/*c*	Mo *K*α radiation, λ = 0.71073 Å
*a* = 12.3270 (7) Å	Cell parameters from 4475 reflections
*b* = 13.3114 (7) Å	θ = 3.0–25.4°
*c* = 7.9673 (4) Å	µ = 3.94 mm^−^^1^
β = 100.091 (2)°	*T* = 296 K
*V* = 1287.13 (12) Å^3^	Block, green
*Z* = 2	0.26 × 0.20 × 0.18 mm

### Data collection

**Table:**

Bruker APEXII CCD diffractometer	1844 reflections with *I* > 2σ(*I*)
φ and ω scans	*R*_int_ = 0.054
Absorption correction: multi-scan (*SADABS*; Bruker, 2014)	θ_max_ = 25.6°, θ_min_ = 3.0°
*T*_min_ = 0.549, *T*_max_ = 0.745	*h* = −14→14
15951 measured reflections	*k* = −16→16
2391 independent reflections	*l* = −9→9

### Refinement

**Table:**

Refinement on *F*^2^	Primary atom site location: structure-invariant direct methods
Least-squares matrix: full	Hydrogen site location: inferred from neighbouring sites
*R*[*F*^2^ > 2σ(*F*^2^)] = 0.054	H-atom parameters constrained
*wR*(*F*^2^) = 0.152	*w* = 1/[σ^2^(*F*_o_^2^) + (0.0854*P*)^2^ + 2.577*P*] where *P* = (*F*_o_^2^ + 2*F*_c_^2^)/3
*S* = 1.04	(Δ/σ)_max_ < 0.001
2391 reflections	Δρ_max_ = 0.96 e Å^−^^3^
170 parameters	Δρ_min_ = −1.29 e Å^−^^3^
73 restraints	

### Special details

**Table:**

Geometry. All e.s.d.'s (except the e.s.d. in the dihedral angle between two l.s. planes) are estimated using the full covariance matrix. The cell e.s.d.'s are taken into account individually in the estimation of e.s.d.'s in distances, angles and torsion angles; correlations between e.s.d.'s in cell parameters are only used when they are defined by crystal symmetry. An approximate (isotropic) treatment of cell e.s.d.'s is used for estimating e.s.d.'s involving l.s. planes.

### Fractional atomic coordinates and isotropic or equivalent isotropic displacement parameters (Å^2^)

**Table:**

	*x*	*y*	*z*	*U*_iso_*/*U*_eq_	Occ. (<1)
Ru1	0.5000	0.5000	0.0000	0.0438 (2)	
Cl1	0.58971 (14)	0.46179 (12)	0.28373 (18)	0.0584 (4)	
Br1	−0.03134 (9)	0.59834 (15)	0.3013 (2)	0.1661 (8)	
N1	0.6046 (4)	0.6219 (4)	−0.0113 (6)	0.0497 (11)	
N2	0.4128 (4)	0.6192 (4)	0.0781 (6)	0.0503 (11)	
C7	0.3075 (5)	0.6159 (5)	0.1302 (8)	0.0563 (14)	
C8	0.3001 (6)	0.5804 (5)	0.2904 (8)	0.0631 (16)	
H8	0.3635	0.5596	0.3632	0.076*	
C6	0.4524 (6)	0.7073 (5)	0.0614 (8)	0.0600 (16)	
H6	0.4143	0.7650	0.0817	0.072*	
C5	0.5584 (5)	0.7125 (4)	0.0100 (8)	0.0555 (14)	
C9	0.2001 (7)	0.5755 (6)	0.3439 (10)	0.079 (2)	
H9	0.1950	0.5530	0.4528	0.094*	
C3	0.7127 (7)	0.8027 (6)	−0.0497 (11)	0.080 (2)	
H3	0.7478	0.8626	−0.0679	0.096*	
C4	0.6092 (7)	0.8027 (5)	−0.0099 (10)	0.075 (2)	
H4	0.5736	0.8630	0.0035	0.090*	
C1	0.7073 (6)	0.6224 (5)	−0.0410 (10)	0.0713 (19)	
H1	0.7433	0.5616	−0.0482	0.086*	
C10	0.1078 (7)	0.6047 (8)	0.2316 (13)	0.095 (3)	
C11B	0.113 (3)	0.624 (4)	0.061 (3)	0.087 (7)	0.50 (9)
H11B	0.0484	0.6332	−0.0171	0.105*	0.50 (9)
C2	0.7629 (7)	0.7123 (6)	−0.0620 (11)	0.082 (2)	
H2	0.8344	0.7102	−0.0844	0.099*	
C12B	0.2127 (19)	0.630 (4)	0.010 (4)	0.078 (7)	0.50 (9)
H12B	0.2170	0.6422	−0.1035	0.093*	0.50 (9)
C12A	0.2157 (19)	0.661 (3)	0.035 (6)	0.077 (7)	0.50 (9)
H12A	0.2222	0.6925	−0.0674	0.092*	0.50 (9)
C11A	0.116 (3)	0.660 (4)	0.087 (5)	0.095 (8)	0.50 (9)
H11A	0.0561	0.6956	0.0276	0.114*	0.50 (9)

### Atomic displacement parameters (Å^2^)

**Table:**

	*U*^11^	*U*^22^	*U*^33^	*U*^12^	*U*^13^	*U*^23^
Ru1	0.0540 (4)	0.0368 (3)	0.0405 (4)	0.0017 (3)	0.0078 (3)	0.0008 (3)
Cl1	0.0767 (10)	0.0531 (8)	0.0423 (7)	0.0036 (8)	0.0016 (6)	0.0044 (6)
Br1	0.0811 (7)	0.2433 (19)	0.1885 (15)	0.0388 (9)	0.0645 (8)	0.0555 (13)
N1	0.061 (3)	0.043 (3)	0.046 (3)	−0.002 (2)	0.011 (2)	−0.002 (2)
N2	0.061 (3)	0.048 (3)	0.041 (2)	0.004 (2)	0.008 (2)	0.000 (2)
C7	0.063 (3)	0.053 (3)	0.054 (3)	0.015 (3)	0.012 (3)	0.001 (3)
C8	0.063 (4)	0.074 (4)	0.052 (4)	0.011 (3)	0.010 (3)	0.008 (3)
C6	0.073 (4)	0.041 (3)	0.065 (4)	0.007 (3)	0.011 (3)	−0.001 (3)
C5	0.067 (4)	0.041 (3)	0.057 (3)	0.000 (3)	0.006 (3)	0.005 (3)
C9	0.078 (5)	0.090 (5)	0.072 (5)	0.005 (4)	0.026 (4)	0.007 (4)
C3	0.080 (5)	0.057 (4)	0.102 (6)	−0.015 (4)	0.013 (4)	0.004 (4)
C4	0.081 (5)	0.046 (4)	0.097 (6)	−0.003 (4)	0.014 (4)	0.010 (3)
C1	0.069 (4)	0.051 (4)	0.097 (5)	−0.001 (3)	0.024 (4)	−0.006 (3)
C10	0.063 (4)	0.123 (7)	0.106 (6)	0.029 (5)	0.033 (4)	0.023 (5)
C11B	0.064 (7)	0.112 (18)	0.084 (8)	0.034 (12)	0.009 (8)	0.014 (10)
C2	0.067 (4)	0.079 (5)	0.105 (6)	−0.018 (4)	0.026 (4)	−0.006 (4)
C12B	0.069 (8)	0.099 (19)	0.064 (9)	0.028 (10)	0.010 (5)	0.019 (11)
C12A	0.071 (8)	0.077 (16)	0.081 (12)	0.015 (9)	0.010 (7)	0.027 (11)
C11A	0.061 (7)	0.111 (19)	0.111 (12)	0.016 (12)	0.009 (10)	0.037 (12)

### Geometric parameters (Å, º)

**Table:**

Ru1—Cl1	2.3908 (14)	C5—C4	1.376 (9)
Ru1—Cl1^i^	2.3907 (14)	C9—H9	0.9300
Ru1—N1	2.084 (5)	C9—C10	1.374 (12)
Ru1—N1^i^	2.084 (5)	C3—H3	0.9300
Ru1—N2^i^	2.073 (5)	C3—C4	1.367 (11)
Ru1—N2	2.073 (5)	C3—C2	1.365 (11)
Br1—C10	1.895 (8)	C4—H4	0.9300
N1—C5	1.356 (8)	C1—H1	0.9300
N1—C1	1.328 (8)	C1—C2	1.403 (10)
N2—C7	1.432 (8)	C10—C11B	1.392 (17)
N2—C6	1.286 (8)	C10—C11A	1.391 (17)
C7—C8	1.379 (9)	C11B—H11B	0.9300
C7—C12B	1.387 (15)	C11B—C12B	1.365 (17)
C7—C12A	1.385 (15)	C2—H2	0.9300
C8—H8	0.9300	C12B—H12B	0.9300
C8—C9	1.374 (10)	C12A—H12A	0.9300
C6—H6	0.9300	C12A—C11A	1.364 (17)
C6—C5	1.438 (9)	C11A—H11A	0.9300
			
Cl1^i^—Ru1—Cl1	180.0	C4—C5—C6	122.0 (6)
N1—Ru1—Cl1	91.11 (14)	C8—C9—H9	121.0
N1^i^—Ru1—Cl1	88.89 (14)	C10—C9—C8	118.0 (7)
N1^i^—Ru1—Cl1^i^	91.11 (14)	C10—C9—H9	121.0
N1—Ru1—Cl1^i^	88.89 (14)	C4—C3—H3	121.0
N1—Ru1—N1^i^	180.0 (2)	C2—C3—H3	121.0
N2^i^—Ru1—Cl1^i^	93.28 (13)	C2—C3—C4	118.0 (7)
N2^i^—Ru1—Cl1	86.71 (13)	C5—C4—H4	120.4
N2—Ru1—Cl1^i^	86.72 (13)	C3—C4—C5	119.3 (7)
N2—Ru1—Cl1	93.29 (13)	C3—C4—H4	120.4
N2^i^—Ru1—N1^i^	76.9 (2)	N1—C1—H1	119.1
N2—Ru1—N1	76.9 (2)	N1—C1—C2	121.7 (7)
N2^i^—Ru1—N1	103.1 (2)	C2—C1—H1	119.1
N2—Ru1—N1^i^	103.1 (2)	C9—C10—Br1	119.0 (7)
N2—Ru1—N2^i^	180.0	C9—C10—C11B	120.9 (15)
C5—N1—Ru1	114.3 (4)	C9—C10—C11A	121.3 (16)
C1—N1—Ru1	128.9 (4)	C11B—C10—Br1	119.4 (15)
C1—N1—C5	116.9 (5)	C11A—C10—Br1	117.9 (17)
C7—N2—Ru1	127.4 (4)	C10—C11B—H11B	120.0
C6—N2—Ru1	116.1 (4)	C12B—C11B—C10	120 (3)
C6—N2—C7	116.0 (5)	C12B—C11B—H11B	120.0
C8—C7—N2	119.3 (5)	C3—C2—C1	120.4 (7)
C8—C7—C12B	120.0 (17)	C3—C2—H2	119.8
C8—C7—C12A	118.5 (18)	C1—C2—H2	119.8
C12B—C7—N2	119.5 (15)	C7—C12B—H12B	120.7
C12A—C7—N2	121.5 (17)	C11B—C12B—C7	119 (3)
C7—C8—H8	119.6	C11B—C12B—H12B	120.7
C9—C8—C7	120.8 (6)	C7—C12A—H12A	119.3
C9—C8—H8	119.6	C11A—C12A—C7	121 (3)
N2—C6—H6	121.5	C11A—C12A—H12A	119.3
N2—C6—C5	117.0 (6)	C10—C11A—H11A	121.4
C5—C6—H6	121.5	C12A—C11A—C10	117 (3)
N1—C5—C6	114.5 (5)	C12A—C11A—H11A	121.4
N1—C5—C4	123.5 (6)		
			
Ru1—N1—C5—C6	8.8 (7)	C8—C7—C12B—C11B	11 (4)
Ru1—N1—C5—C4	−173.2 (6)	C8—C7—C12A—C11A	−8 (4)
Ru1—N1—C1—C2	173.2 (6)	C8—C9—C10—Br1	179.8 (7)
Ru1—N2—C7—C8	76.6 (7)	C8—C9—C10—C11B	10 (3)
Ru1—N2—C7—C12B	−91 (3)	C8—C9—C10—C11A	−16 (3)
Ru1—N2—C7—C12A	−113 (3)	C6—N2—C7—C8	−111.0 (7)
Ru1—N2—C6—C5	−7.1 (8)	C6—N2—C7—C12B	81 (3)
Br1—C10—C11B—C12B	179.5 (19)	C6—N2—C7—C12A	59 (3)
Br1—C10—C11A—C12A	−178 (2)	C6—C5—C4—C3	176.3 (7)
N1—C5—C4—C3	−1.4 (11)	C5—N1—C1—C2	−4.5 (11)
N1—C1—C2—C3	1.0 (13)	C9—C10—C11B—C12B	−10 (4)
N2—C7—C8—C9	−179.5 (7)	C9—C10—C11A—C12A	18 (5)
N2—C7—C12B—C11B	178.6 (18)	C4—C3—C2—C1	2.6 (13)
N2—C7—C12A—C11A	−178 (2)	C1—N1—C5—C6	−173.1 (6)
N2—C6—C5—N1	−1.3 (9)	C1—N1—C5—C4	4.8 (10)
N2—C6—C5—C4	−179.3 (6)	C10—C11B—C12B—C7	0 (4)
C7—N2—C6—C5	179.7 (6)	C2—C3—C4—C5	−2.3 (12)
C7—C8—C9—C10	1.5 (12)	C12B—C7—C8—C9	−12 (3)
C7—C12A—C11A—C10	−6 (4)	C12A—C7—C8—C9	10 (3)

Symmetry code: (i) −*x*+1, −*y*+1, −*z*.

### Hydrogen-bond geometry (Å, º)

**Table:**

*D*—H···*A*	*D*—H	H···*A*	*D*···*A*	*D*—H···*A*
C8—H8···Cl1^ii^	0.93	2.79	3.472 (7)	132
C6—H6···Cl1^iii^	0.93	2.83	3.673 (7)	151
C3—H3···Br1^iv^	0.93	3.13	3.797 (8)	131
C4—H4···Cl1^v^	0.93	2.94	3.529 (7)	122

Symmetry codes: (ii) −*x*+1, −*y*+1, −*z*+1; (iii) −*x*+1, *y*+1/2, −*z*+1/2; (iv) *x*+1, −*y*+3/2, *z*−1/2; (v) *x*, −*y*+3/2, *z*−1/2.
